# Bioprosthetic Valve Fracturing in Valve‐in‐Valve TAVI: Clinical and Echocardiographic Outcomes in Failing Perimount Aortic Bioprostheses—A Multicenter Registry

**DOI:** 10.1002/ccd.31686

**Published:** 2025-06-19

**Authors:** Hendrik Ruge, Melchior Burri, Julia Schreyer, Teodora‐Cristiana Georgescu, Derk Frank, Won‐Keun Kim, Ole de Backer, Martin Beyer, Andreas Schäfer, Chiara Fraccaro, Giuseppe Tarantini, Erion Xhepa, Michael Joner, Markus Krane, Héctor Alfonso Alvarez Covarrubias

**Affiliations:** ^1^ Department of Cardiovascular Surgery, Institute Insure, German Heart Center Munich, School of Medicine & Health Technical University of Munich Munich Germany; ^2^ German Center for Cardiovascular Research (DZHK) partner side Hamburg/Kiel/Lübeck Kiel Germany; ^3^ Department of Internal Medicine III, Cardiology and Critical Care University Hospital Schleswig‐Holstein, Campus Kiel Kiel Germany; ^4^ Med. Clinic I, Cardiology & Angiology University of Giessen und Marburg GmbH Giessen Germany; ^5^ Department of Cardiology Kerckhoff Heart Center Bad Nauheim Germany; ^6^ The Heart Center, Rigshospitalet Copenhagen Denmark; ^7^ University Heart and Vascular Center Hamburg Hamburg German; ^8^ Department of Cardiac, Thoracic and Vascular Science University Medical School of Padua, Interventional Cardiology Unit Padova Italy; ^9^ Department of Cardiology, German Heart Center Munich, School of Medicine & Health Technical University of Munich Munich Germany; ^10^ Department of Surgery, Division of Cardiac Surgery Yale School of Medicine New Haven USA; ^11^ DZHK (German Center for Cardiovascular Research)—partner site Munich Heart Alliance Munich Germany

**Keywords:** bioprostheic valve fracturing, hemodynamics, valve‐in‐valve TAVI

## Abstract

**Background:**

Data comparing clinical and hemodynamic outcomes of bioprosthetic valve fracturing (BVF) and “standard”‐postdilatation during valve‐in‐valve transcatheter heart valve implantation (ViV‐TAVI) are lacking. The authors aimed to analyze hemodynamic and clinical outcomes of BVF compared to “standard”‐postdilatation during ViV‐TAVI.

**Methods:**

The REDUCE registry included patients who underwent ViV‐TAVI within a Perimount surgical aortic valve bioprosthesis (Edwards Lifesciences, USA). Procedures were categorized to no postdilatation, “standard”‐postdilatation and BVF. Hemodynamic and clinical outcomes at 30 days were collected and compared. A linear regression model was built to predict mean aortic gradient after ViV‐TAVI.

**Results:**

A total of 240 patients from six European sites were included. Median age was 78 years [IQR 70; 83], logistic EuroSCORE calculated 20.0%[IQR 12.2; 33.1] and 159 patients (66%) were male. One hundred fourty‐four Perimount valves (60%) had a true internal diameter (ID) ≤ 21 mm. Self‐expanding valves (SEV) and ballon‐expandable valves (BEV) were used in 60% and 40% of cases, respectively. One hundred sixteen procedures (48%) were executed without postdilatation, in 88 procedures (37%) “standard”‐postdilatation and in 36 procedures (15%) BVF was used. 30‐day survival was 93.3%. VARC‐3 device success at 30 days was 71%. A multivariable regression analysis of the mean aortic gradient after ViV‐TAVI showed a significant association with surgical valve size (−0.84 mmHg, *p* = 0.001; per 1 mm surgical valve size increase), execution of postdilatation (−3.25 mmHg, *p* = 0.007) and type of transcatheter heart valve (SEV: −7.31 mmHg, *p* < 0.001).

**Conclusions:**

When performing ViV‐TAVI within a Perimount surgical aortic bioprosthesis with a true ID ≤ 21 mm, the hemodynamic valve performance is most optimal when implanting a SEV‐TAV and when postdilating the TAV‐in‐SAV complex. BVF did not result in superior hemodynamics compared to “standard”‐postdilatation.

**Abbreviations**
BEV: balloon‐expandable valve
BVF: bioprosthetic valve fracturing
ID: inner diameter
NYHA: New York heart association
PPM: prosthesis‐patient mismatch
SAVR: surgical aortic valve replacement
SEV: self‐expanding valve
THV: transcatheter heart valve
VARC: valve academic research consortium
ViV‐TAVI: valve‐in‐valve transcatheter aortic valve implantation

## 1 Introduction

Valve‐in‐valve transcatheter aortic valve implantation (ViV‐TAVI) is a well‐established therapy for the treatment of failed surgical bioprostheses in patients with higher operative risk [1, 2]. Postprocedural improvements in NYHA functional class remain stable, with > 85% of patients after ViV‐TAVI being in NYHA functional class I or II at 3‐year follow‐up [3, 4]. For small surgical valves (i.e., labeled size ≤ 21 mm or inner diameter ≤ 20 mm), postoperative mean transprosthetic gradients after ViV‐TAVI have been shown to be significantly higher compared to larger surgical valves [5, 6]. For failed small surgical valves, worse 1‐ and 8‐year survival is reported after ViV‐TAVI compared to patients with larger failed surgical valves [6, 7]. Conflicting data are reported regarding postoperative transprosthetic gradients > 20 mmHg in association with 1‐year mortality after ViV‐TAVI [8, 9]. Bioprosthetic valve fracturing (BVF) aims to improve transprosthetic gradients in ViV‐TAVI. Bench‐test studies identified surgical valves responsive to BVF and established protocols to perform successful BVF [10, 11, 12, 13]. The reduction of gradients following BVF in ViV‐TAVI [14, 15, 16, 17] remains stable at 1 year follow up [15, 18]. Currently, there is only limited data available comparing transprosthetic gradients after ViV‐TAVI with BVF and without BVF [16, 19]. We report on transprosthetic gradients immediately after ViV‐TAVI with or without BVF focusing on one dedicated surgical valve type to minimize confounding. Furthermore, we sought to assess the effect of BVF on transprosthetic gradient after ViV‐TAVI by building a multivariable regression model.

## 2 Methods

This retrospective multicenter study was conducted at six European sites. All patients undergoing ViV‐TAVI for a failing Edwards Perimount aortic bioprosthesis model P 2900 were identified from each institutional TAVI database. Ethics committee approval was gained (31/22 S‐NP).

### 2.1 Valve‐in‐Valve TAVI Procedure

The access route for ViV‐TAVI was reported. Transcatheter heart valves (THV) were categorized into self‐expanding or balloon‐expandable valve types. Procedures were categorized to no postdilatation, “standard”‐postdilatation and BVF.

### 2.2 BVF

BVF was performed at the operator's discretion. In brief, postdilatation with a high‐pressure, non‐compliant balloon (True Dilatation balloon or Atlas Gold balloon, C.R. Bard, Murray Hill, USA), at least 1 mm larger than the true ID of the surgical valve was performed under rapid ventricular pacing. A high pressure indeflator was used to fill the balloon with diluted contrast followed by continuous pressure increase until BVF was achieved. Successful bioprosthetic ring fracturing was defined as the combined occurrence of a sudden drop in indeflator pressure (eventually accompanied by an audible bump) and the disappearance of the balloon waist with achievement of a fully cylindrical shape.

### 2.3 Endpoints

The primary endpoint was mean transprosthetic gradient at 30‐day follow‐up. Secondary endpoints were VARC‐3 defined technical success at exit from the procedure room, VARC‐3 defined device success at 30‐day [20], VARC‐3 defined vascular and bleeding complications, perioperative stroke, need for permanent pacemaker implantation, anular rupture, coronary obstruction and aortic dissection.

### 2.4 Data Collection

Demographics, procedural details, intra‐hospital course, and adverse events were prospectively recorded and reported according to the VARC‐3 recommendations [20]. Choice of THV size was determined using the internal diameter of the failing Perimount valve obtained from the preoperative contrast‐enhanced computerized tomography and after consulting the valve‐in‐valve App (Valve in Valve app, UBQO and Dr. Bapat).

### 2.5 Statistical Analysis

Statistical analysis was conducted using R statistical software language (version 3.6.1, R Foundation for Statistical Computing, Vienna, Austria). Continuous variables with normal distribution are presented as mean ± standard deviation. Continuous variables without normal distribution are presented as median (IQR) and as percentages for categorical data. Comparison between groups was performed using either a Fisher exact test for binominal variables, *t*‐test for continuous and normally distributed variables and a Wilcox Rank‐sum test for continuous variables with non‐parametric distribution. A *p* value of < 0.05 was considered as significant.

A multivariable regression model based on surgical valve size and failure mode, execution of postdilatation or BVF and type of THV was built to estimate mean aortic valve gradient after ViV‐TAVI. Patients with labeled valve size ≤ 25 were included in the model. Q−Q plotting was used to assess normal distribution of the variables (Supporting Information S1: Figure S1). Fitted value plots of the residuals were assessed for heteroskedasticity (Supporting Information S1: Figure S2). F‐statistics was used to assess the overall significance of the model. R‐squared was used to measure the strength of the relationship between the model and the dependent variable.

## 3 Results

A total of 240 patients from six European sites undergoing ViV‐TAVI for a failing Perimount P 2900 were included in the REDUCE registry (Biop**R**osthetic valv**E** fracturing **DU**ring valve in valve trans**C**atheter aortic valve implantation for failing P**E**rimount bioprosthesis Registry). Median age was 78 years [IQR 70; 83], logistic EuroSCORE calculated 20.0% [IQR 12.2; 33.1] and 159 patients (66%) were male. Table 1 displays the baseline characteristics of the total cohort and the three subgroups. Baseline characteristics were balanced between the groups with exception of baseline serum‐creatinine.

**Table 1:** Baseline clinical characteristics.

*n*	All	No postdilatation	BVF	“Standard” postdilatation	*p* value
	240	116	36	88	
Male sex, *n* (%)	159 (66)	86 (74)	19 (53)	54 (61)	0.029
Age, year (IQR)	78 [70−83]	78 [69−83]	78 [72−81]	79 [70−83]	1.0
Body Mass Index, kg/m^2^ (IQR)	26 [23−29]	26 [23−29]	27 [24−30]	25 [22−30]	0.3
CAD, *n* (%)	151 (63)	74 (64)	20 (56)	57 (65)	0.6
Previous CABG, *n* (%)	68 (28)	33 (28)	7 (19)	28 (32)	0.7
PAD, *n* (%)	38 (16)	19 (16)	6 (17)	13 (15)	1.0
Hypertension, *n* (%)	206 (86)	99 (85)	30 (83)	77 (88)	0.8
Diabetes mellitus, *n* (%)	51 (21)	25 (22)	11 (31)	15 (17)	0.2
Previous stroke, *n* (%)	24 (10)	13 (11)	6 (17)	5 (6)	0.3
COPD, *n* (%)	33 (14)	17 (15)	4 (11)	12 (14)	0.9
Creatinin mg/dL, mean (IQR)	1.1 [0.9−1.4]	1.2 [0.9−1.6]	1.0 [0.8−1.4]	1.0 [0.9−1.3]	0.028
Previous pacemaker, *n* (%)	46 (19)	28 (24)	5 (14)	13 (15)	0.2
Atrial fibrillation, *n* (%)	81 (34)	37 (32)	11 (31)	33 (38)	0.6
Porcelain aorta	15 (6.2%)	5 (4.3%)	4 (11.1%)	6 (6.8%)	0.2
STS PROM, % (IQR)	3.6 [2.1−5.7]	3.5 [2.0−6.0]	3.5 [2.4−4.6]	3.6 [1.7−4.8]	0.8
Euroscore 2, % (IQR)	7.6 [4.3‐14.0]	7.9 [4.5−15.2]	8.4 [4.6−14.7]	6.8 [4.1−10.8]	0.4
Log. Euroscore, % (IQR)	20.0 [12.2‐33.1]	21.0 [12.0−33.2]	22.0 [14.4−30.8]	17.0 [13.3−33.0]	0.6

Mean time between surgical aortic valve replacement (SAVR) and ViV‐TAVI was 11.7 ± 4.0 years. Failure mode was stenosis (42%), regurgitation (10%) and mixed disease (48%). The proportion of cases with inner diameter ≤ 21 mm was 60% (*n* = 144). Table 2 displays baseline echocardiography data.

**Table 2:** Baseline echocardiography characteristics and details on failing SAVR.

	All	No postdilatation	BVF	“Standard” postdilatation	
*n*	240	116	36	88	*p* value
LVEF, % (IQR)	55.0 [20.0−60.0]	55.0 [20.0−64.0]	59.5 [22.0−65.0]	55.0 [20.0−72.0]	0.3
Aortic valve peak gradient, mmHg (IQR)	65 [45−83]	60 [41−75]	67 [48−86]	73 [56−90]	0.002
Aortic valve mean gradient, mmHg (IQR)	38 [27−50]	34 [24−46]	44 [27−53]	42 [33−57]	0.003
Aortic valve regurgitation, *n* (%)					0.6
None	70 (29)	31 (27)	9 (25)	30 (34)	
Mild	71 (30)	30 (26)	12 (33)	29 (33)	
Moderate	37 (15)	19 (16)	7 (19)	11 (13)	
Severe	59 (25)	34 (29)	8 (22)	17 (19)	
Mitral valve regurgitation, *n* (%)					1.0
None	35 (15)	20 (17)	5 (14)	10 (11)	
Mild	93 (39)	50 (43)	13 (36)	30 (34)	
Moderate	25 (10)	11 (10)	5 (14)	9 (10)	
Severe	6 (3)	3 (3)	1 (3)	2 (2)	
Tricuspid valve regurgitation, *n* (%)					0.4
None	55 (23)	26 (22)	8 (22)	21 (24)	
Mild	73 (30)	42 (36)	9 (25)	22 (25)	
Moderate	25 (10)	12 (10)	6 (17)	7 (8)	
Severe	2 (1)	0	1 (3)	1 (1)	
Systolic PAP (mmHg), mean (IQR)	45 [35–56]	45 [35–57]	42 [35–58]	45 [32–53]	0.6
LVOT (mm), mean (IQR)	21 [20.0–23.0]	21 [19.2–23.0]	21 [20.5–22.5]	21 [20.0–23.2]	0.8
LVEDD (mm), mean (SD)	51.0 ± 9.2	53.1 ± 9.4	50.0 ± 7.9	47.8 ± 8.8	0.07
Time to surgical valve failure, years (SD)	11.7 ± 4.0	11.5 ± 3.5	12.2 ± 3.8	11.9 ± 4.7	0.7
SAVR failure mode, *n* (%)					0.063
Stenosis	100 (42)	40 (35)	16 (44)	44 (50)	
Regurgitation	24 (10)	17 (15)	1 (3)	6 (7)	
Mixed disease	115 (48)	58 (50)	19 (53)	38 (43)	
Perimount valve size, *n* (%)					< 0.001
19	6 (3)	1 (1)	3 (8)	2 (2)	
21	55 (23)	17 (15)	16 (44)	22 (25)	
23	83 (35)	40 (35)	12 (33)	31 (35)	
25	63 (26)	35 (30)	4 (11)	24 (27)	
27	25 (10)	15 (13)	1 (3)	9 (10)	
29	7 (3)	7 (6)	0	0	
LCA heigth, mm (SD)	11.2 ± 4.3	11.7 ± 4.8	9.8 ± 4.9	11.1 ± 3.4	0.2
RCA height, mm (SD)	13.7 ± 5.0	13.7 ± 4.7	14.6 ± 6.0	13.4 ± 4.9	0.6

### 3.1 Procedural Details

**Figure 1:**
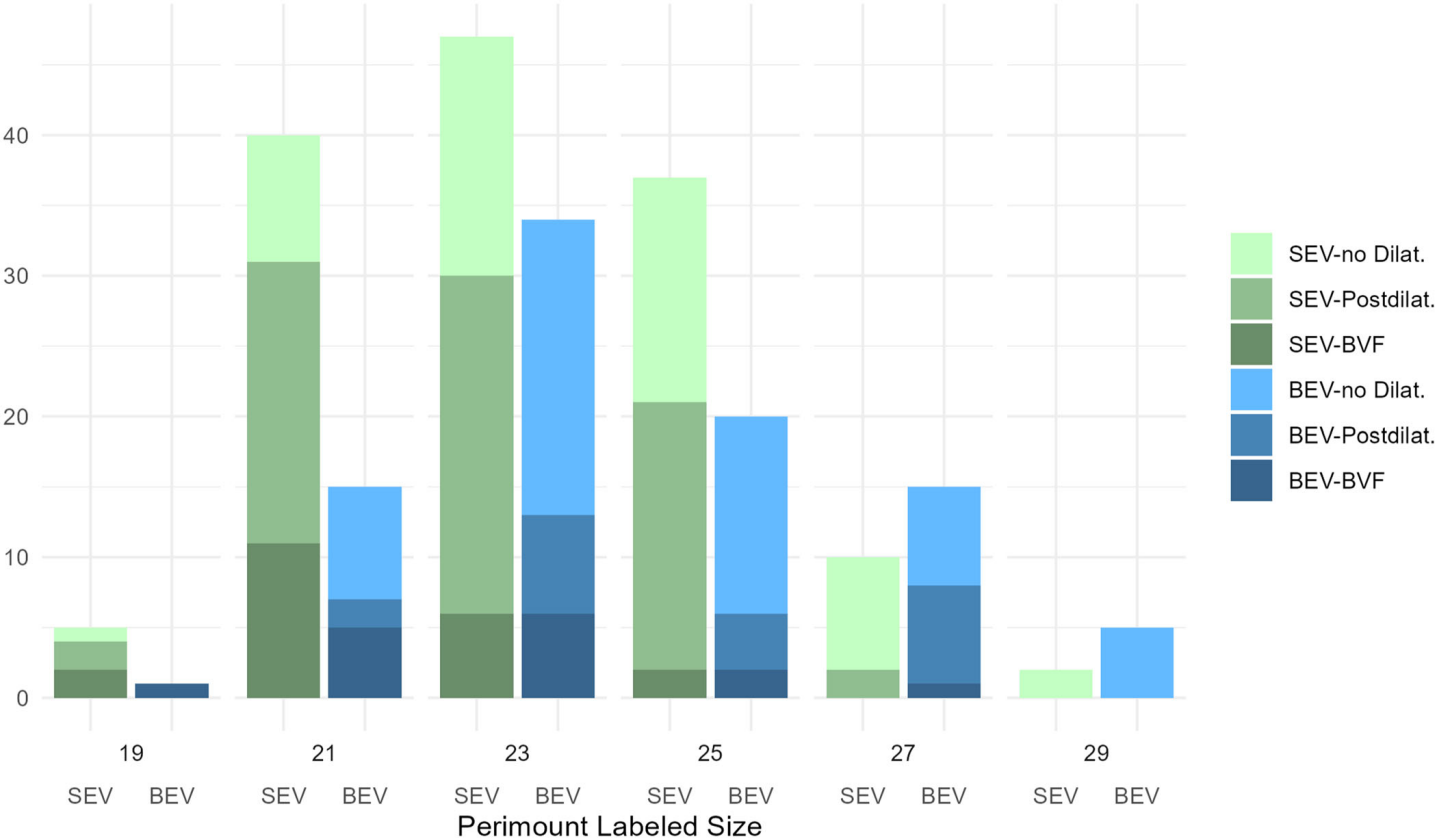
Failing Perimount valves are grouped according to valve size and consecutive implantation of SEV or BEV and execution of “standard”‐postdilatation, BVF or absence of postdilatation. [Color figure can be viewed at wileyonlinelibrary.com]

Table 3 summarizes procedural details and differences among the three groups. ViV‐TAVI was predominantly executed using transfemoral access (93%). Self‐expanding valves were used in 60%, and balloon‐expandable valves were used in 40% of the procedures. One hundred sixteen procedures (48%) were executed without postdilatation, in 88 procedures (37%) “standard”‐postdilatation and in 36 procedures (15%) BVF was executed. Figure 1 displays implantation of SEV or BEV and postdilatation strategy grouped according to the surgical valve size. In all BVF procedures, implantation of the THV was executed first, followed by BVF. VARC‐3 technical success was achieved in 96% of the procedures.

**Table 3:** Procedural characteristics.

	All	No postdilatation	BVF	“Standard” postdilatation	
	240	116	36	88	*p* value
General anaesthesia, *n* (%)	60 (25)	52 (45)	3 (8)	5 (6)	< 0.001
Access, *n* (%)					0.036
Transfemoral	222 (93)	100 (86)	36 (100)	86 (98)	
Apical	13 (5)	12 (10)	0	1 (1)	
Aorta	1 (0.4)	1 (1)	0	0	
Axillary	4 (1.7)	3 (3)	0	1 (1)	
THV Type, *n* (%)					< 0.001
Sapien XT	12 (5)	11 (10)	0	1 (1)	
Sapien 3/3 Ultra	83 (35)	48 (41)	15 (42)	20 (23)	
CoreValve	14 (6)	11 (10)	2 (6)	1 (1)	
EvolutR/Pro	95 (40)	34 (29)	13 (36)	48 (55)	
Acurate	30 (13)	8 (7)	6 (17)	16 (18)	
Portico	6 (3)	4 (3)	0	2 (2)	
deployment mode, *n* (%)					< 0.001
Self‐expanding	145 (60)	57 (49)	21 (58)	67 (76)	
Balloon‐expandable	95 (40)	59 (51)	15 (42)	21 (24)	
THV valve size, *n* (%)					0.036
20	5 (2)	2 (2)	2 (6)	1 (1)	
23	112 (47)	51 (44)	24 (67)	37 (42)	
25	10 (4)	2 (2)	2 (6)	6 (7)	
26	83 (35)	39 (34)	8 (22)	36 (41)	
27	3 (1)	2 (2)	0	1 (1)	
29	25 (10)	18 (16)	0	7 (8)	
34	2 (1)	2 (2)	0	0	
Contrast medium (mL), mean (IQR)	60 [40–94]	58 [40–80]	68 [40−100]	70 [42−107]	0.2
Fluoroscopy time (min), mean (IQR)	14.6 [9.6−21.2]	11.6 [8.3−17.2]	16.8 [10.6−25.4]	16.7 [12.1−21.0]	< 0.001
Procedure time (min), mean (IQR)	58 [46–72]	55 [45–70]	59 [44–72]	63 [48–74]	0.4
Cerebral protect device, *n* (%)	25 (10)	6 (5)	5 (14)	14 (16)	0.035
Chimney stent, *n* (%)					0.055
LCA	3 (1)	1 (1)	1 (3)	1 (1)	
RCA	1 (0.4)	0	1 (3)	0	
both	1 (0.4)	0	1 (3)	0	
BASILICA, *n* (%)					0.058
LCC	1 (0.4)	0	1 (3)	0	
RCC	0	0	0	0	
Pericardial effusion requiring intervention, *n*	0	0	0	0	—
Need for unplanned coronary intervention, *n* (%)	1 (0.4)	1 (1)	0	0	0.6
Need for second THV, *n* (%)	2 (1)	1 (1)	1 (3)	0	0.3
VARC‐3 technical success, *n* (%)	231 (96)	109 (94)	36 (100)	86 (98)	0.2

### 3.2 30‐Day Outcome

**Figure 2:**
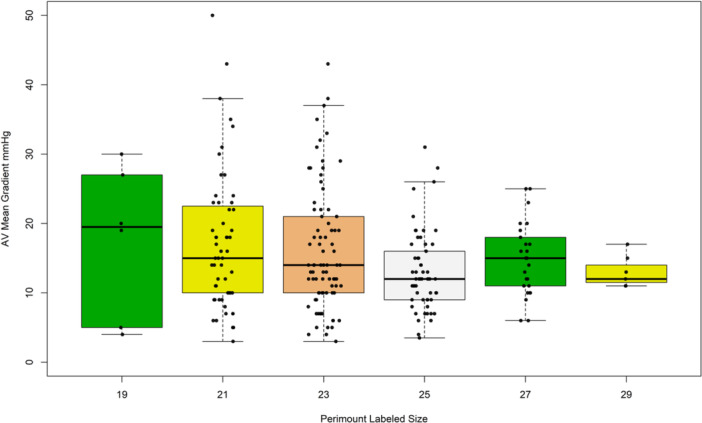
Figure 2 displays 30‐day transprosthetic gradients after ViV TAVI grouped according to the Perimount valve sizes. [Color figure can be viewed at wileyonlinelibrary.com]

**Figure 3:**
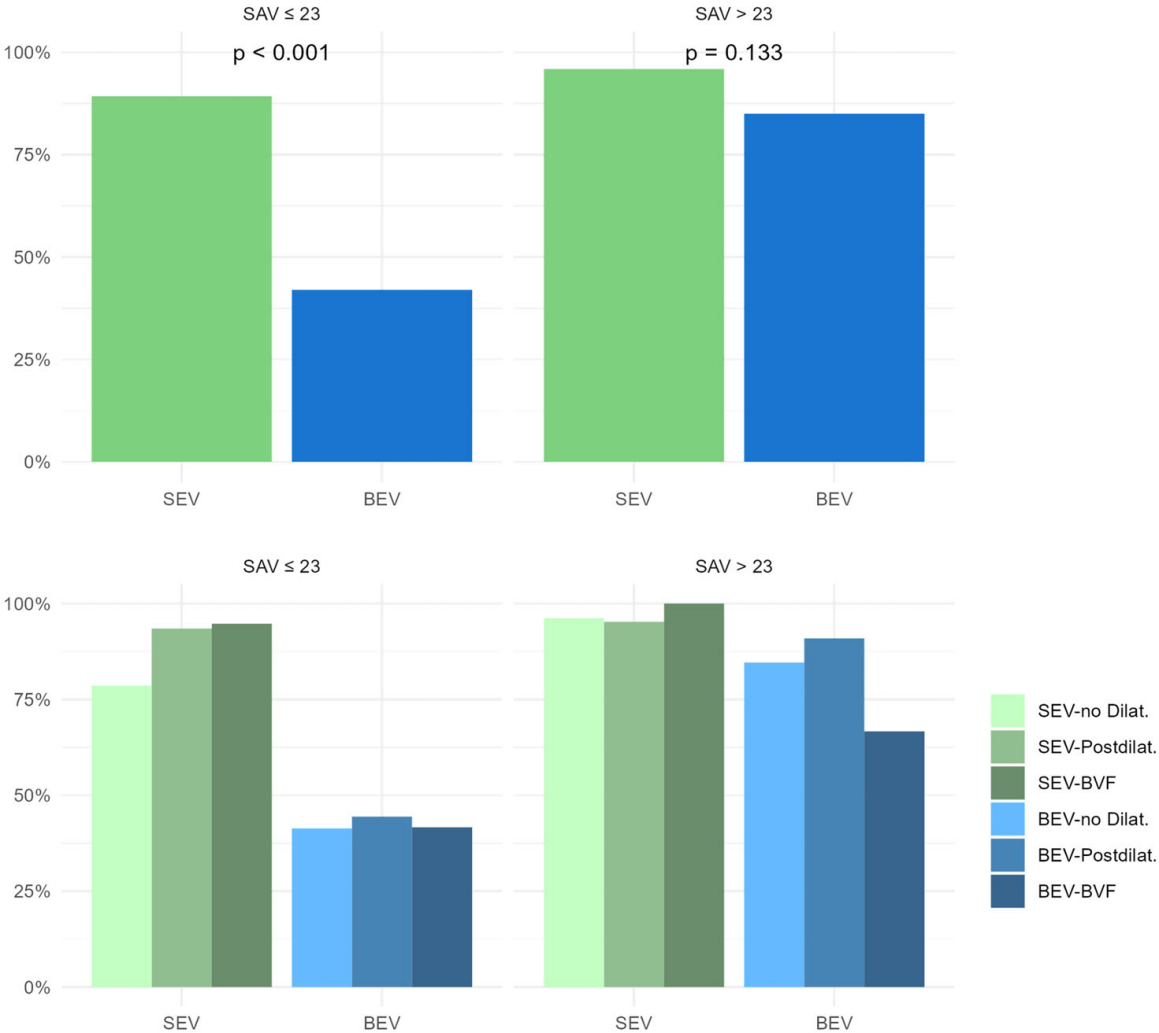
Intended hemodynamic valve performance with aortic mean gradient < 20 mmHg in small and larger Perimount valves with implantation of SEV or BEV and execution of BVF or “standard”‐postdilatation or absence of postdilatation. [Color figure can be viewed at wileyonlinelibrary.com]

30‐day outcome and adverse events are shown in Table 4. None of the patients undergoing BVF suffered a stroke, annular rupture, coronary obstruction or aortic dissection. 30‐day survival was 93.3%. Aortic valve mean gradient measured 15.5 ± 8 mmHg at discharge (*p* < 0.001 against baseline). Aortic valve regurgitation was ≤ mild in 230 procedures (96%). Figure 2 displays 30‐day transprosthetic gradients after ViV‐TAVI grouped according to the Perimount valve sizes. Figure 3 summarizes intended valve performance with aortic valve gradient < 20 mmHg after ViV‐TAVI at 30 days stratified by Perimount valve sizes ≤ 23 and > 23, use of SEV versus BEV, and utilization of BVF, “standard”‐postdilatation or no postdilatation.

**Table 4:** 30‐day outcomes.

	All	No postdilatation	BVF	“Standard” postdilatation	
	240	116	36	88	*p* value
LVEF, % (IQR)	56 [47–60]	55 [40–60]	54 [50–60]	60 [50–60]	0.3
AVA, cm^2^ (SR)	1.68 ± 0.39	1.80 ± 0.46	1.65 ± 0.34	1.56 ± 0.33	0.4
Aortic valve peak gradient, mmHg (IQR)	29 [21−41]	31 [22−41]	32 [29−48]	23 [18−36]	0.023
Aortic valve mean gradient, mmHg (IQR)	16.0 [10.5−22.0]	16.5 [11.8−25.0]	19.0 [14.5−24.5]	12.0 [8.5−19.0]	0.007
AV Regurgitation, *n* (%)					0.09
None/Trace	177 (74)	83 (72)	29 (80)	65 (74)	
Mild	53 (22)	26 (22)	5 (14)	22 (25)	
Moderate	3 (1)	0	2 (6)	1 (1)	
Severe	0	0	0	0	
Renal failure requiring hemodialysis, *n* (%)	1 (0.4%)	0	1 (3%)	0	0.058
Permanent pacemaker requirement, *n* (%)	9 (4)	4 (3)	1 (3)	4 (5)	0.9
VARC‐3 access site vascular complications, *n* (%)					0.2
Major	9 (4)	7 (6)	0	2 (2)	
Minor	7 (3)	3 (3)	2 (6)	2 (2)	
VARC‐3 bleeding complication, *n* (%)					0.4
Life threatening	1 (0.4%)	1 (1)	0	0	
Major	10 (4)	8 (7)	1 (3)	1 (1)	
Minor	1 (0.4%)	1 (1)	0	0	
Stroke					0.3
Fatal	3 (1)	3 (3)	0	0	
Disabling	4 (2)	1 (1)	2 (6)	1 (1)	
NYHA class, *n* (%)					0.3
I	78 (33)	35 (30)	15 (42)	28 (32)	
II	55 (23)	27 (23)	9 (25)	19 (22)	
III	12 (5)	6 (5)	0	6 (7)	
IV	0	0	0	0	
Survival, *n* (%)	224 (93)	106 (91)	34 (94)	84 (96)	0.5
VARC‐3 device success, *n* (%)	172 (72)	76 (66)	26 (72)	70 (80)	0.09

*Note:* For patients without 30‐day echocardiography, predischarge data were included for analysis resulting in missing data of one patient. Eight patients were lost to follow up for 30‐day survival analysis.

**Figure 4:**
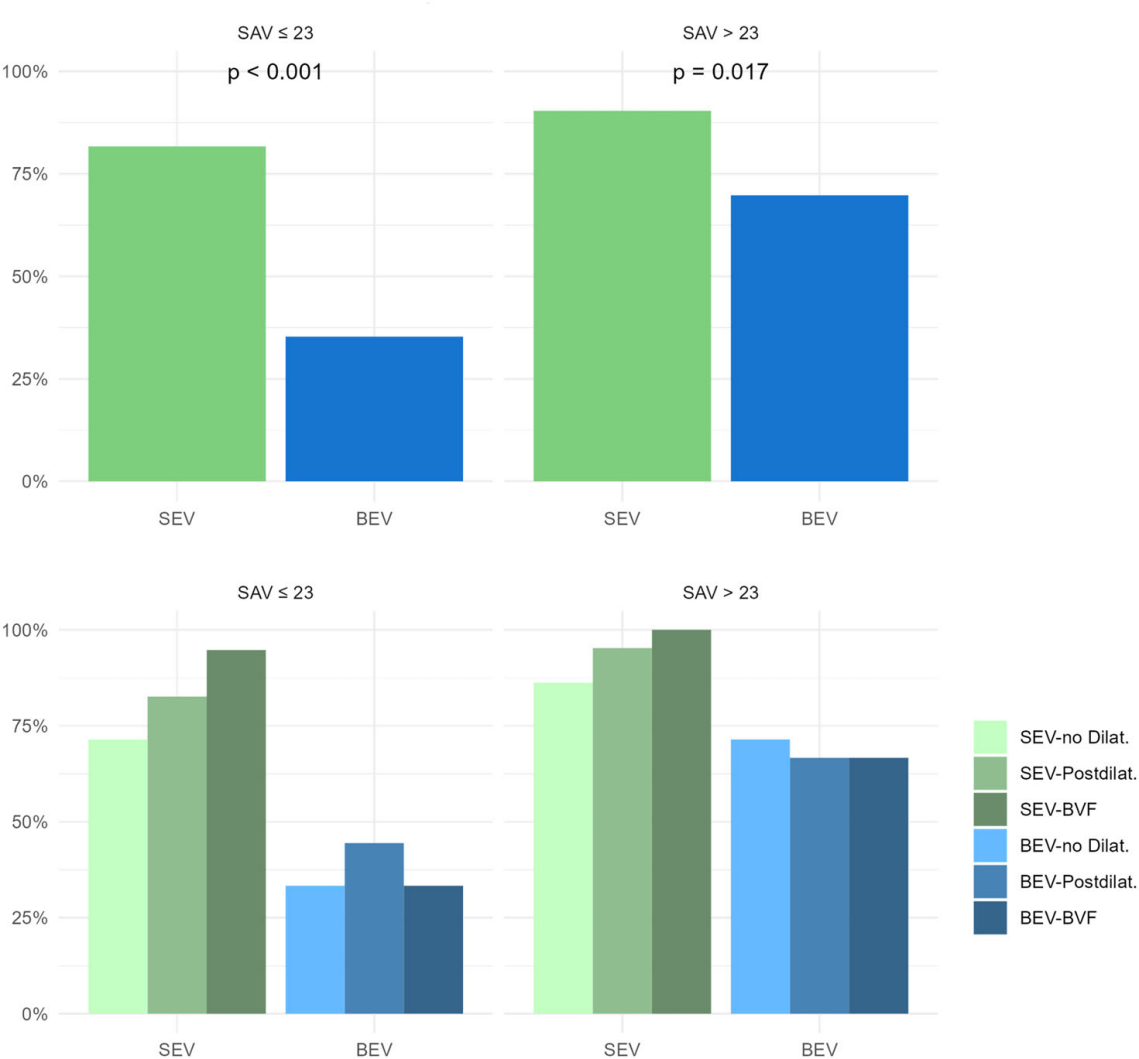
VARC‐3 device success at 30 days in small and larger Perimount valves with implantation of SEV or BEV and execution of BVF or “standard”‐postdilatation or absence of postdilatation. [Color figure can be viewed at wileyonlinelibrary.com]

**Figure 5:**
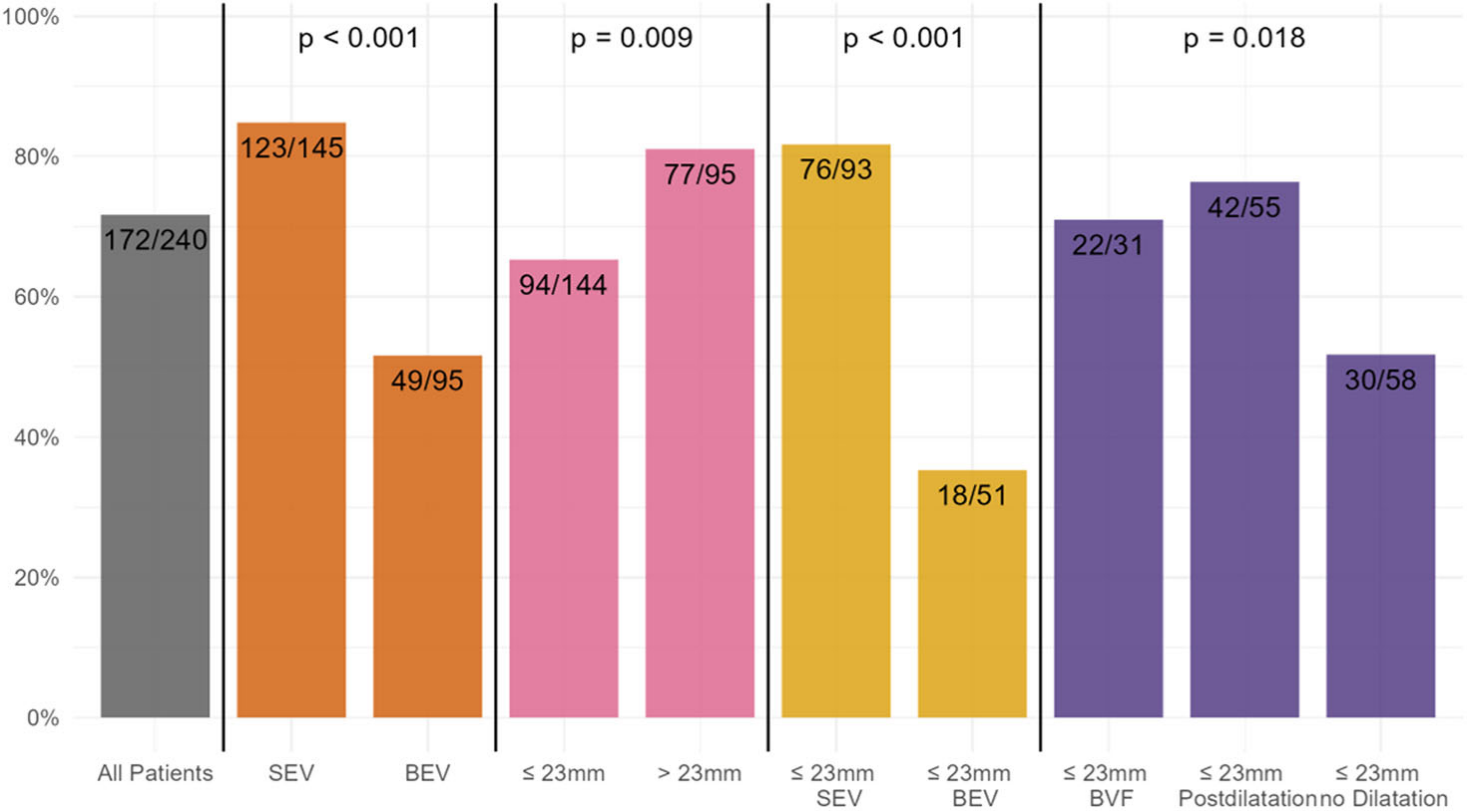
30‐day VARC‐3 device success in SEV and BEV, small and large surgical valves, SEV and BEV in small surgical valves and in small surgical valves applying BVF, “standard”‐postdilatation and no postdilatation. [Color figure can be viewed at wileyonlinelibrary.com]

VARC‐3 device success at 30 days was 71%. Details on VARC‐3 device success comparing SEV and BEV in small (labeled size ≤ 23 mm, i.e., ≤ 21 mm inner diameter) surgical valves (*p* < 0.001) and larger valves (*p* = 0.017) and comparing execution of BVF, “standard”‐postdilatation and no postdilatation in small surgical valves (*p* = 0.018) are displayed in Figures 4 and 5.

### 3.3 Multivariable Regression Analysis

**Figure 6:**
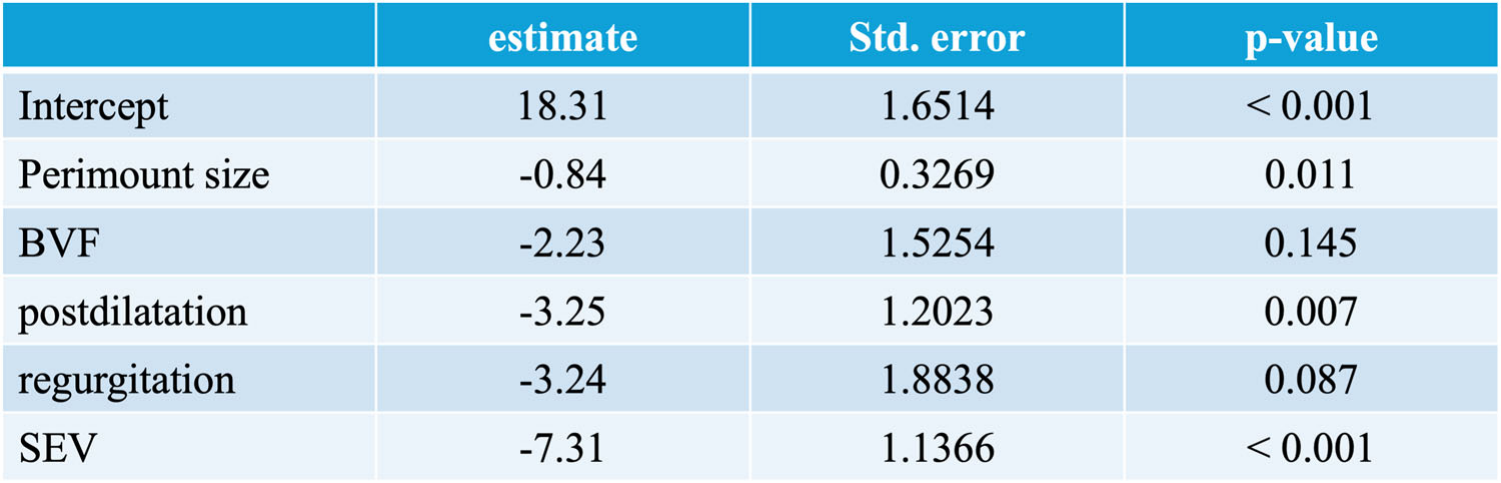
Predictors, parameter estimates, standard error and *p* value for post ViV‐TAVI mean transprosthetic gradient estimation. [Color figure can be viewed at wileyonlinelibrary.com]

A multivariable regression analysis (Central Figure 1, Figure 6) of the mean aortic gradient after ViV‐TAVI showed a significant association with surgical valve size (−0.84 mmHg, *p* = 0.001; per 1 mm surgical valve size increase), execution of postdilatation (−3.25 mmHg, *p* = 0.007) and type of THV (SEV: −7.31 mmHg, *p* < 0.001) (Figure 6). Execution of BVF (−2.23, *p* = 0.14) and failure mode “valvular regurgitation” (−3.24, *p* = 0.09) were not significantly associated with mean aortic gradient after ViV‐TAVI. *p* value of F‐statistics was highly significant (*p* = 7.049e‐10). R‐squared was 0.2698, adjusted R‐squared was 0.2509.

**Central Figure 1:**
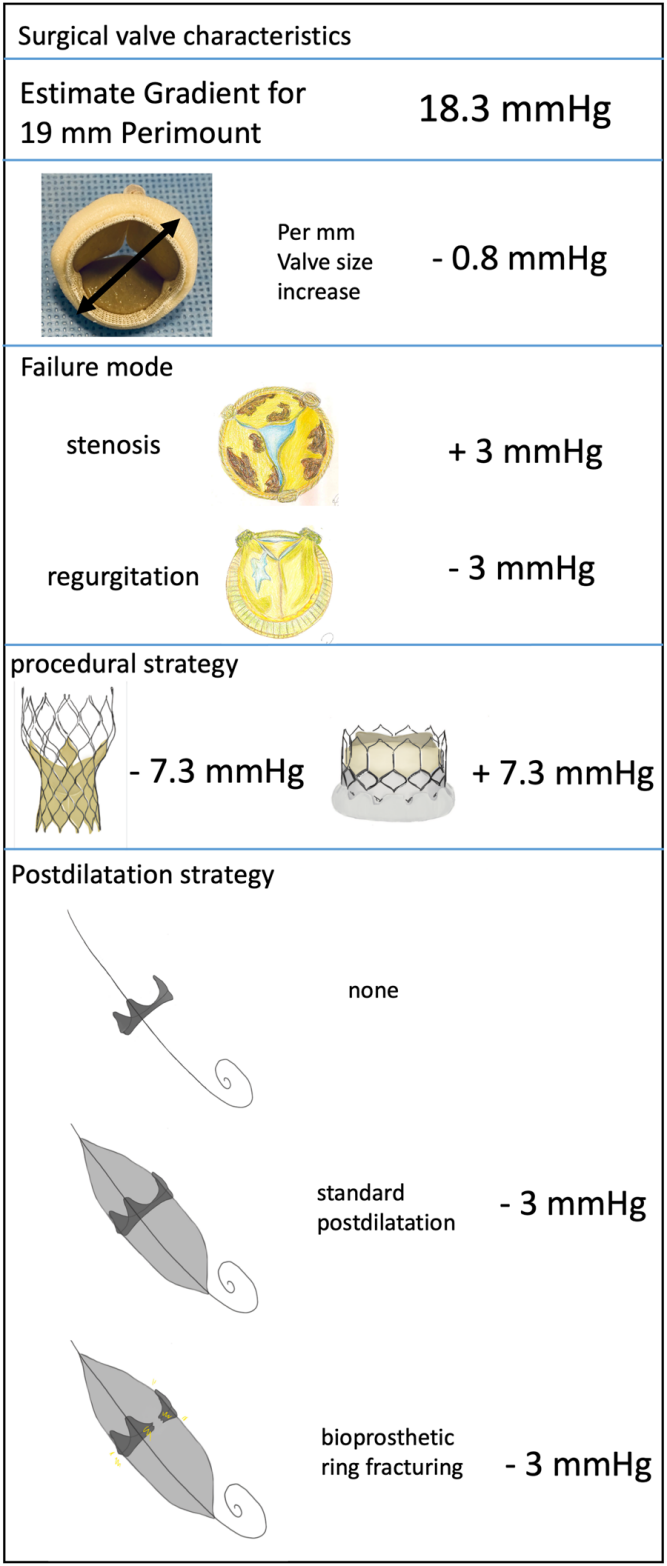
A multivariale regression model predicts post‐ViV mean aortic gradient based on Perimount valve size, failure mode, type of transcatheter heart valve and postdilatation strategy based on an estimate gradient of 18.3 mmHg for a failing 19 mm Perimount valve. [Color figure can be viewed at wileyonlinelibrary.com]

## 4 Discussion

The REDUCE registry is an international multicenter evaluation of ViV‐TAVI procedures focusing on one dedicated surgical valve to homogenize baseline characteristics of the failing surgical valve, thereby improving data interpretation. The key findings of the study are:

- VARC‐3 device success at 30 days was higher in large surgical valves compared to small surgical valves.
- VARC‐3 device success was higher with SEV than with BEV with a 2.3‐fold higher success rate in small surgical valves.
- In a multivariable regression model, lower discharge mean aortic gradient was associated with (a) increasing surgical valve size, (b) use of a self‐expanding THV and (c) “standard”‐postdilatation.

In addition to our main findings, we also found that technical success (96%) and 30‐day survival (96%) were high in ViV‐TAVI. Fatal/disabling stroke, vascular and bleeding complications were rare. BVF did not improve hemodynamic outcome compared to “standard”‐postdilatation in ViV‐TAVI.

### 4.1 Hemodynamic Outcomes

In line with our findings, previous studies reported higher aortic gradients after ViV‐TAVI for failing small as compared to larger surgical valves [5, 6, 7]. Presence of small surgical bioprostheses has been shown to independently correlate with poorer clinical outcomes at mid‐ to long‐term follow‐up [6]; treatment of this patient subgroup remains challenging due to high rates of patient‐prosthesis mismatch (PPM) as well as persistently increased transvalvular gradients. Elevated mean transvalvular gradients (> 20 mmHg) represented the predominant driver of failed achievement of the composite VARC‐3 device success endpoint in the present cohort. In addition to surgical bioprosthesis size, type of THV being implanted has been shown to have a profound impact on post‐procedural transvalvular gradients and need for re‐intervention, particularly in small pre‐existing bioprostheses [6]. The prospective, randomized LYTEN trial showed that patients receiving a SEV displayed significantly lower transvalvular gradients as well as significantly higher rates of intended valve performance (BEV: 30% vs. SEV: 76%; *p* < 0.001) at 1‐year follow‐up [9]. Interestingly, our results closely mirror those of the LYTEN trial, thereby adding further evidence supporting the superiority of SEV as compared to BEV being implanted in small surgical bioprostheses, in terms of device success rates (BEV: 35% in the present study vs. 30% in the LYTEN trial; SEV: 82% in the present study vs. 76% in the LYTEN trial) [9].

### 4.2 BVF

In an attempt to overcome the issue of PPM and suboptimal THV expansion as well as the resulting high postprocedural transvalvular gradients and the potentially negative impact on valve durability following ViV procedures, BVF has been introduced [10, 14, 15, 21]. Interims hemodynamic data between THV implantation and BVF confirmed transvalvular gradient reduction in a small case series [11]. However, previous studies reporting on BVF either included only BVF patients [14, 18], compared ViV‐TAVI procedures with BVF to ViV‐TAVI procedures without postdilatation [15] or focused on a single THV [17]. Furthermore, inclusion of multiple surgical valve types resulted in heterogenous patient cohorts making the hemodynamic outcomes difficult to interpret [14, 16, 18, 19]. The REDUCE registry is the first comparison of BVF and “standard”‐postdilatation in a reasonably large patient population undergoing ViV procedures for degenerated Edwards Perimount P 2900 bioprostheses.

The 21% attempted BVF rate reported from the STS/TVT registry [17] is comparable to the frequency of BVF utilization in the present study. With small surgical valves having a higher risk for elevated gradient after ViV‐TAVI, our findings support previous reports that BVF is predominantly executed in surgical valves displaying an inner diameter ≤ 21 mm [15, 18]. A subgroup analysis of 1085 patients including 250 attempted BVF from the STS/TVT registry with information on the failing surgical valve size showed, that attempted BVF resulted in lower gradients at discharge and 30‐day follow‐up in patients with surgical valves ≤ and >21 mm inner diameter [17],

The optimal timing of BVF (before or after THV Implantation) has been a matter of debate, with theoretical advantages of the former strategy represented by limited damage to the THV leaflets and favorable impact on valve durability, while the latter strategy has the potential advantages of reducing the risk of acute aortic insufficiency and hemodynamic compromise as well as of achieving an optimal THV expansion [22]. Both, preclinical bench studies and clinical investigations have shown that BVF performed after THV implantation is associated with better THV expansion, less pinwheeling and lower transvalvular gradients [22, 23] without apparent deleterious effects on leaflet integrity [24]. In line with these findings, in the present study BVF was performed following THV implantation in all cases. Additionally, selection of balloon size for BVF represents an important consideration. In a retrospective study, use of a non‐compliant balloon at least 3 mm larger than the true inner diameter was an independent predictor of a lower post‐procedural transvalvular gradient [14].

In line with our findings, lower mean aortic gradients were found with BVF compared to ViV‐TAVI without postdilatation [14, 15, 16, 17]. Building a linear regression model, we showed a significant association of “standard”‐postdilatation with gradient reduction. Gradient reduction was also shown for BVF. Nevertheless, statistical significance was not reached maybe due to the smaller sample size. Future prospective randomized trials are warranted to further evaluate a potential hemodynamic benefit of BVF over “standard”‐postdilatation.

### 4.3 Limitations

Due to the retrospective design, the study has several limitations:

1. The retrospective nature of the study introduces inherent biases. This limitation affects the ability to establish causal relationships and may influence the results. Various factors such as access routes (e.g., transfemoral, transapical), types and generations of THV, different SAVR failure modes (stenosis, regurgitation, and mixed disease), and operator discretion in performing BVF introduce potential confounders. While we attempted to account for these variables in our multivariable regression model, residual confounding may still be present.
2. BVF was performed at the operator's discretion without a standardized protocol across all sites. Transvalvular gradients before and after execution of BVF were not prospectively recorded. This lack of uniformity in the execution of BVF may lead to variations in outcomes. Future studies should consider implementing a dedicated protocol for BVF to ensure consistency and comparability of results.
3. The sample size for the BVF group was relatively small (15% of the total cohort). This limited sample size reduces the statistical power to detect differences and increases the margin of error. Larger, prospective studies are needed to validate our findings and further explore the benefits of BVF.
4. Clinical and echocardiographic data as well as successful BVF were reported by individual sites and not adjudicated by a central core laboratory. This could lead to variability in data quality and interpretation. Standardizing data collection and involving core laboratories in future studies would improve data accuracy and reliability.
5. There may be a selection bias in deciding whether or not to perform BVF, as it was left to the operator's discretion. Patients who underwent BVF might have had different baseline characteristics compared to those who did not, potentially influencing the outcomes. Efforts were made to balance baseline characteristics, but unmeasured confounders might still affect the results.

## 5 Conclusion

When performing ViV‐TAVI within a Perimount surgical aortic bioprosthesis with a true ID < 21 mm, the hemodynamic valve performance is most optimal when implanting a SEV‐TAV and when postdilating the TAV‐in‐SAV complex. BVF did not result in superior hemodynamics compared to “standard”‐postdilatation.

## Ethics Statement

The study was approved by the local ethical committee Ethics (31/22 S‐NP). For retrospective studies, patient informed consent is not required per local authorities and local ethical committee regulations.

## Conflicts of Interest

Dr. Ruge serves as a physician proctor for Abbott and Edwards Lifescience, is consultant for Medtronic, Abbott and Edwards Lifescience and is member of the Abbott advisory board. Dr. Krane is a physician proctor and a member of the medical advisory board for Sanamedi, a physician proctor for Peter Duschek, is a medical consultant for EVOTEC and Moderna and has received speakers' honoraria from EDWARDS, AtriCure, Medtronic and Terumo.

Dr. Kim is a proctor for Abbott, Boston Scientific, Meril Life Sciences, received honoraria from Abbott, Boston Scientific, Edwards Lifesciences, JenaValve, Meril Life Sciences, is advisory board member to Boston Scientific, HID Imaging, and received institutional fees from Boston Scientific. Dr. Beyer received travel compensation from Abbott. Dr. De Backer received institutional research grants and consulting fees from Abbott, Boston Scientific, Medtronic and SMT. Dr. Frank is a proctor for Medtronic and Edwards Lifesciences, received honoraria from Abbott, Boston Scientific, Edwards Lifesciences and Medtronic and received a research grant from Edward Lifesciences. Dr Schäfer is a proctor for Abbott, received speaker honoraria and travel compensation from Abbott, Boston Scientific and Edwards Lifesciences. Dr. Xhepa reports consulting services for Edwards Lifesciences, lecture fees ans honoraria from AstraZeneca, Boston Scientific and SIS Medical not related to the current work; proctor fees from Abbott Vascular and financial support for attending meetings and/or travel expenses from SIS Medical and Abbott Vascular. Dr. Alvarez Covarrubias received lecture fees from SIS Medical, LifeTech and Edwards Lifesciences, travel support from LifeTech and Abbott and proctor fees from LifeTech. Professor Joner reports personel fees from Abbott, Alchimedics S.A.S, AstraZeneca, Biotronik, Cardiac Dimensions, Edwards Lifesciences, Medtronic, Recor, Shockwave, TriCares, Veryan and grants and personel fees from Boston Scientific aboutside the submitted work.

## Data Avaiablity Statement

Data are available on request.

## Supporting information

Supplemental Figure 1. Q‐Q plot indicating normal distribution of variables included in regression model.

Supplemental Figure 2. Heteroskedasticity testing. Plot indicates an equal variance of residuals over the range of measured values.
