# Supplementary figures and images for: Bioprosthetic Valve Fracturing in Valve‐in‐Valve TAVI: Clinical and Echocardiographic Outcomes in Failing Perimount Aortic Bioprostheses—A Multicenter Registry
Source: Catheter Cardiovasc Interv. 2025 Jun 19;106(2):1409–20. doi: 10.1002/ccd.31686 (PMC12336782; doi:10.1002/ccd.31686)

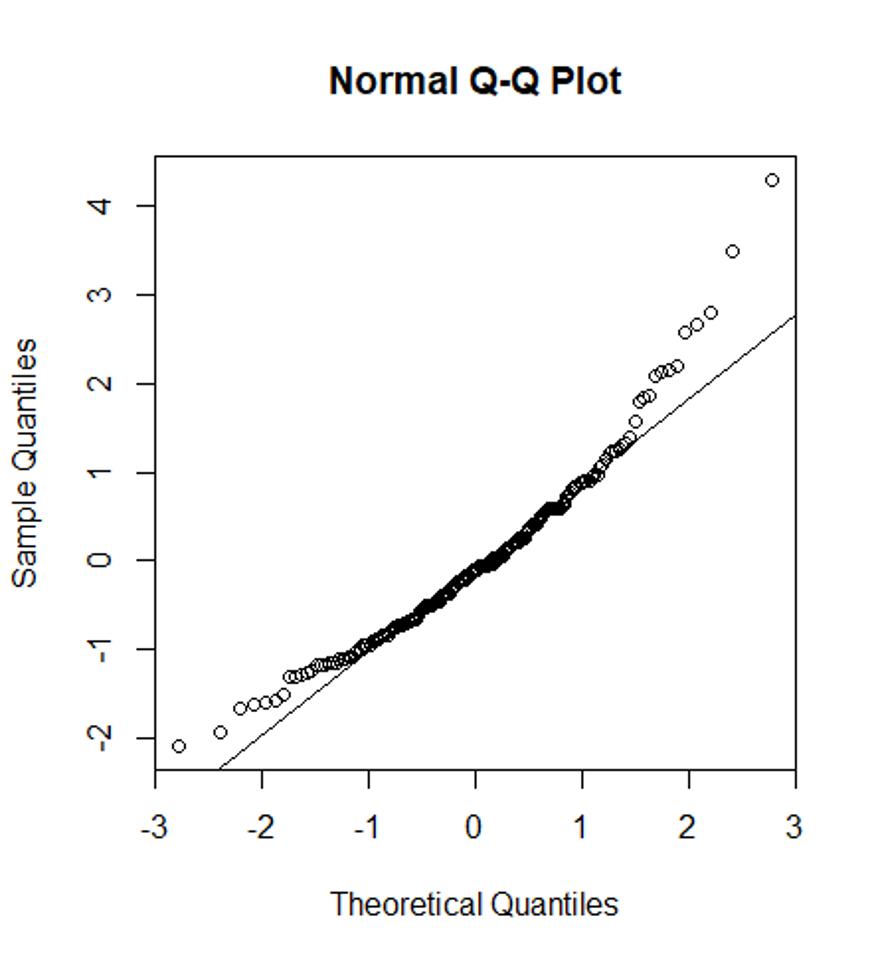

Supplement: Supplementary file 1 — Supplemental Figure 1. Q‐Q plot indicating normal distribution of variables included in regression model. [file CCD-106-1409-s001.tiff]

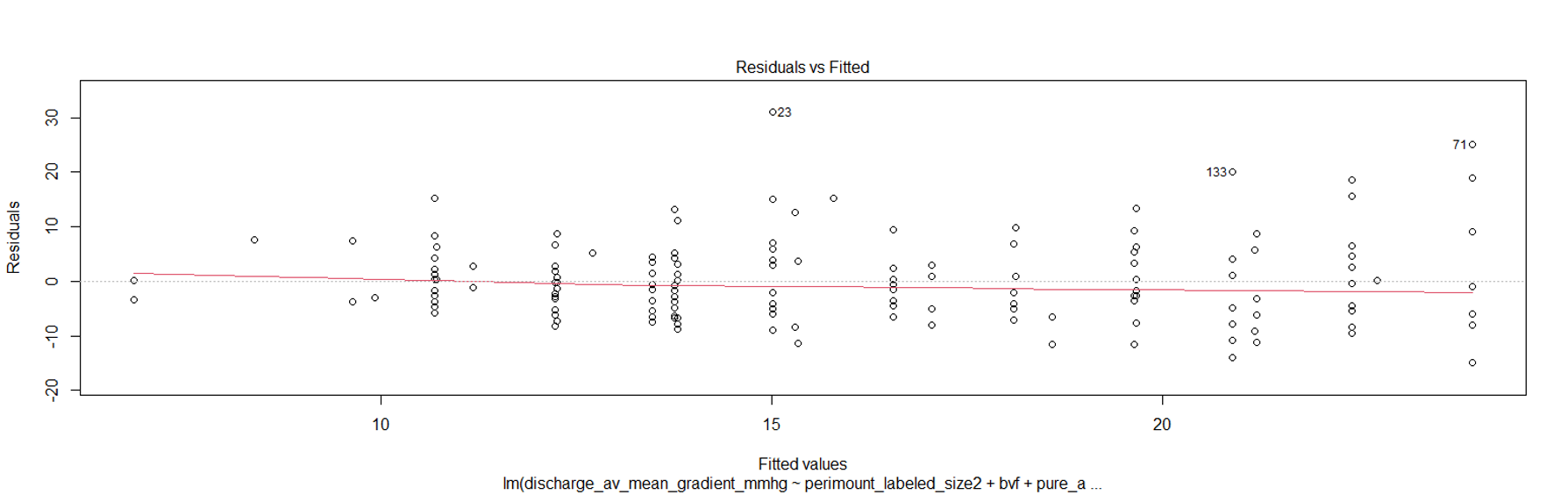

Supplement: Supplementary file 2 — Supplemental Figure 2. Heteroskedasticity testing. Plot indicates an equal variance of residuals over the range of measured values. [file CCD-106-1409-s002.tiff]
